# High‐resolution episcopic microscopy enables three‐dimensional visualization of plant morphology and development

**DOI:** 10.1002/pld3.161

**Published:** 2019-11-06

**Authors:** Yuval Cinnamon, Olga Genin, Yiftah Yitzhak, Joseph Riov, Israel David, Felix Shaya, Anat Izhaki

**Affiliations:** ^1^ Institute of Animal Science Volcani Center, Agricultural Research Organization Rishon LeZion Israel; ^2^ Institute of Plant Sciences Volcani center, Agricultural Research Organization Rishon LeZion Israel; ^3^ The Robert H. Smith Institute of Plant Sciences and Genetics in Agriculture, The Robert H. Smith Faculty of Agriculture, Food and Environment The Hebrew University of Jerusalem Rehovot Israel

**Keywords:** 3D modeling, adventitious roots, citrus, HREM, persimmon rootstock, secretory cavity

## Abstract

The study of plant anatomy, which can be traced back to the seventeenth century, advanced hand in hand with light microscopy technology and relies on traditional histologic techniques, which are based on serial two‐dimensional (2D) sections. However, these valuable techniques lack spatial arrangement of the tissue and hence provide only partial information. A new technique of whole‐mount three‐dimensional (3D) imaging termed high‐resolution episcopic microscopy (HREM) can overcome this obstacle and generate a 3D model of the specimen at a near‐histological resolution. Here, we describe the application of HREM technique in plants by analyzing two plant developmental processes in woody plants: oil secretory cavity development in citrus fruit and adventitious root formation in persimmon rootstock cuttings. HREM 3D models of citrus fruit peel showed that oil cavities were initiated schizogenously during the early stages of fruitlet development. Citrus secretory cavity formation, shape, volume, and distribution were analyzed, and new insights are presented. HREM 3D model comparison of persimmon rootstock clones, which differ in their rooting ability, revealed that difficult‐to‐root clones failed to develop adventitious roots due to their inability to initiate root primordia.

**Significance Statement**

We describe the application of a technique for whole‐mount three‐dimensional imaging termed high‐resolution episcopic microscopy (HREM) in plants. Analysis of secretory cavity development and adventitious root formation demonstrated the potential use of the HREM application for studying developmental processes in plants.

## 1 INTRODUCTION

Traditional histological techniques using serial two‐dimensional (2D) sections which are stained and visualized under light microscopy were already applied in the seventeenth century to study plant morphology and anatomy (Bolam, Jones, & Paton, 1973). These methods enabled high‐resolution analysis of tissue structure at the cellular level and therefore had a pivotal contribution to the study of plant anatomy. However, 2D sections can provide only limited information, since they completely lack the spatial arrangement of the tissue. To confront these constraints, different approaches for computer‐based three‐dimensional (3D) reconstruction of histological sections by sequential section realigning were developed (Andreasen, Drewes, Assentoft, & Larsen, 1992; Fiala, 2005; Guest & Baldock, 1995). However, these techniques are laborious, prone to alignment errors, and result in 3D models with lower resolution quality compared with the 2D sections, partially due to the effect of tissue sectioning and staining (Mohun & Weninger, 2011). To overcome these obstacles, a new technique for whole‐mount 3D imaging termed high‐resolution episcopic microscopy (HREM) was developed (Geyer, Maurer‐Gesek, Reissig, & Weninger, 2017; Geyer et al., 2014; Mohun & Weninger, 2011; Weninger et al., 2014, 2006). HREM is a microscope–microtome‐based imaging system, which generates hundreds to thousands of perfectly aligned thin‐section images of an embedded tissue. A fluorescence stereomicroscope and a digital camera facilitate a block‐face image capture of fluorescent dyes mixture, which stains the embedded specimen in the block. Following every section (typically ~ 1–5 µm thick), the camera captures the image of the block cut surface. The images are stacked and processed using 3D visualization software to generate a 3D model of the specimen at a near‐histological resolution (~2 µm^3^ per voxel). The 3D model can then be virtually sectioned in any plane and enables metric analysis of the visualized structures (Geyer et al., 2017). Since its first introduction (Weninger & Mohun, 2007), the HREM method was used in various medical applications, such as morphological analysis of model organisms and human tissue visualization (Gershon et al., 2018; Geyer et al., 2014, 2015; Henkelman, Friedel, Lerch, Wilson, & Mohun, 2016; Mohun & Weninger, 2011; Pokhrel, Ben‐Tal Cohen, Genin, Sela‐Donenfeld, & Cinnamon, 2017; Weninger et al., 2014). The HREM method was shown to yield 3D images with higher resolution compared with high‐resolution X‐ray computed tomography (HRXCT), magnetic resonance imaging (MRI), and optical projection tomography (OPT) (Geyer et al., 2017; Geyer, Mohun, & Weninger, 2009). In computerized tomography‐based imaging systems, such as OPT and HRXCT, the raw data are a set of projections which are then processed by an algorithm to create virtual sections that are later used for 3D reconstruction, leading to potential artifacts given this indirect multistep process. In contrast, the data set generated by HREM for 3D modeling are the actual images of thousands of perfectly aligned sections, thus obviating the need for virtualization and overriding artifacts generated by computerized tomography algorithms. Though HREM fails to reach confocal, light sheet, or electron microscopy resolutions, it can provide 3D imaging of much larger specimens (effectively up to 10 × 10 × 15 mm) (Geyer et al., 2017, 2009). Despite the wide and growing use of HREM in 3D imaging of animal model organisms and human tissues, this technique has not yet been applied in plants. Here, we describe the application of the HREM technique in studying two plant developmental processes: oil cavity development in citrus fruit peel and adventitious root formation in persimmon cuttings.

Citrus essential oils, which consist mainly of volatile monoterpenoid and sesquiterpenoid compounds, accumulate in specialized secretory cavities occurring in the stem, leaves, flower organs (except the stamens), and fruit (Knight, Klieber, & Sedgley, 2001). Biosynthesis of the essential oil compounds was suggested to take place in the epithelial cells lining the cavity with subsequent secretion into the cavity lumen (Voo, Grimes, & Lange, 2012). Anatomical studies of citrus secretory cavities using 2D sections were carried out to characterize cavity formation and development. Cell divisions in the epidermal and sub‐epidermal layers initiate the formation of the secretory cavity (Liang, Wu, Lun, & Lu, 2006). At the next stage, a lumen is formed at the center of the secretory cavity, which gradually expands and accumulates essential oils. Studies of the mechanism leading to the formation of the central cavity began more than a hundred years ago, but remain controversial to this day. Several studies and most text books have described the formation of the secretory cavity as a lysigenous process, which involves cell wall degradation to initiate lumen formation (Esau, 1977; Fahn, 1988; Turner, 1999). Other studies have shown that the cavities are initiated schizogenously by cell wall separation (knight et al., 2001; Liang et al., 2006; Thomson, Platt‐Aloia, & Endress, 1976). Cavity formation by overlapping schizogenous and lysigenous events was also described in *Citrus sinesis* and *Citrus limon* secretory cavities (Bennici & Tani, 2004). However, it was suggested that cell lysigeny observed during cavity formation was an artifact of the tissue fixation which resulted in poor tissue preservation (Turner, 1999). Citrus secretory cavities are initiated in the flavedo, the pigmented region of the pericarp, at the early stages of fruitlet development and reach their final structure in the immature green fruit (Bennici & Tani, 2004; Knight et al., 2001; Voo et al., 2012). During fruit maturation, secretory cavities continue to expand and gain a spherical or a pyriform shape (Bennucu & Tani, 2004; Knight et al., 2001; Liang et al., 2006; Voo et al., 2012). To estimate the essential oil amount in citrus fruit glands, secretory cavity volumes and density were calculated using 2D sections (Voo et al., 2012). Significant variability in the cavity volumes was found during all stages of fruit development. It was suggested that the differences in the secretory cavity volumes reflect continuous development and expansion of the cavities (Voo et al., 2012). However, the variability in the cavity volumes may stem from the limited spatial information gained from the 2D sections, which may lead to inaccurate calculations. We utilized the HREM 3D imaging to study *Citrus limon* secretory cavity anatomy during different stages of fruit development. Citrus oil cavity formation, shape, volume, density, and distribution were analyzed, and new insights regarding their development are presented.

Development of adventitious roots from stem cutting is an important trait in herbaceous and woody plants, which provides a powerful tool for clonal propagation (Haissig & Riemenschneider, 1988; Hartman, Kester, Davies, & Geneve, 2014). The process of adventitious root formation contains four stages: 1. Cell differentiation; 2. Cell division; 3. Formation of root primordia from the dividing cells; and 4. Root elongation and emergence (Davies, Lazarte, & Joiner, 1982; De Klerk, Keppel, Brugge, & Meekes, 1995). The ability to form adventitious roots is affected by various factors, namely the ontogenic stage of the mother plant and the clone genotype (Haissig & Riemenschneider, 1988; Hartmannet al., 2014). Histological studies of adventitious root formation in cuttings demonstrated that root primordia are originated in many woody plants from the secondary phloem adjacent to the cambium layer (Bellini, Pacurar, & Perrone, 2014; Hartmann et al., 2014; Izhaki et al., 2018; Naija, Elloumi, Jbir, Ammar, & Kevers, 2008). Comparative anatomical studies of rooting and non‐rooting cuttings were carried out to decipher the basis for poor rooting ability. Some studies attributed the inhibition of adventitious root development in difficult‐to‐root cuttings to the presence of an anatomical barrier manifested by a lignified sclerenchyma layer, which was suggested to act as a physical barrier preventing adventitious root emergence (Beakbane, 1961; Edwards & Thomas, 1980; Goodin, 1965), while other studies showed no correlation between lignified sclerenchyma and low rooting ability (Davies et al., 1982; Sachs, Loreti, & Bie, 1964). Several studies suggested that the inhibition of adventitious root formation was related to the capacity of the tissue to initiate root primordia rather than to the presence of an anatomical barrier (Amissah, Paolillo, & Bassuk, 2008; Davies & Hartmann, 1988; White & Lovell, 1984). Thus far, 2D histological analyses have been applied to characterize the anatomical basis of poor rooting ability (Amissah et al., 2008; Ballester, San‐José, Vidal, Fernández‐Lorenzo, & Vieitez, 1999; Harbage, Stimart, & Evert, 1993; Porfírio, Gomes da Silva, Cabrita, Azadi, & Peixe, 2016). However, the intrinsic limitation of 2D serial sections, which provide only sporadic characterization of the tissue along the cutting base, has encumbered these attempts. To study the relationship between stem anatomy and rooting ability, easy‐ and difficult‐to‐root persimmon (*Dyospyros virginiana*) rootstock clones (Izhaki et al., 2018) were analyzed by HREM and detailed 3D histology images of the cuttings were generated. Comparison of the 3D models revealed that difficult‐to‐root clones failed to develop adventitious roots due to their inability to initiate root primordia.

## 2 MATERIALS AND METHODS

### 2.1 Citrus growth conditions

*Citrus limon* fruits at different developmental stages were collected from twenty‐year‐old trees grown in an orchard at the Volcani Center, ARO, Rishon LeZion, Israel.

### 2.2 Persimmon rootstock growth

*Dyospyros virginiana* genotypes were grown in the field under intensive irrigation and fertilization and used as mother plants for cuttings collection. Difficult‐to‐root genotypes were grafted on seedling scions. The mother plants were pruned in February at a height of about 40 cm above the ground level. Shoots of the current year growth were used as the source of cuttings for the rooting experiments.

### 2.3 Rooting of cuttings

Rooting of cuttings was performed as described by Izhaki et al., (2018). Briefly, Shoots were collected from the mother plants early in the morning, wrapped in a moist filter paper, and placed in plastic bags in a cooler until cutting preparation. Sub‐apical semi‐hardwood cuttings having 3–4 nodes and 2–3 mm in diameter were used. The leaves close to the base of the cuttings were removed, and the remaining upper leaves were cut in half. Before rooting, the cuttings were treated by dipping for 2 min in a solution containing 0.3% merpan (Adama), 0.25% sportak (Gadot‐Agro), and 0.05% Triton X‐100 (Adama) to prevent inoculation by pathogenic fungi. The base of the cuttings was dipped in a solution containing 6,000 mg/L indole‐3‐butyric acid potassium salt (K‐IBA), (Sigma) for 10 s. Rooting was performed in a cooled and shaded (70% shade) greenhouse, in which the air temperature was maintained at 25–29°C. The cuttings were rooted on rooting beds containing vermiculite No. 3: crushed polystyrene (1:1, v\v), with intermittent mist applied every 15 min for 10 s. 10‐ to 15‐mm stem fragments were taken from the cutting base and sectioned by HREM.

### 2.4 Sample preparation and embedding

Sample preparation and embedding were performed as described by Geyer et al. (2017). Briefly, tissues were fixed in 4% paraformaldehyde (w/v) under vacuum for 1–2 hr at room temperature and then incubated overnight at 4°C. Fixed tissues were rinsed twice with phosphate buffered saline and dehydrated through a graded series of ethanol (30%, 50%, 70%, 80%, and 95%). The dehydrated tissues were placed in an infiltration solution containing catalyzed monomer A of the JB‐4 (2‐hydroxyethyl methacrylate) embedding kit (Polysciences), 0.275% w/v Eosin B (Sigma‐Aldrich), and 0.056% w/v Acridine orange (Sigma‐Aldrich). Samples were vacuumed for 2–3 hr, infiltration solution was replaced with a fresh solution, and the samples were incubated at 4°C on rotating shaker for 10 days (full solution infiltration is achieved within 5–10 days, according to the sample size, which ranges from 3–10 mm in diameter). Tissues were embedded in embedding solution containing the JB‐4 infiltration solution and JB4‐solution B, according to manufacturer guidelines. Samples were placed in a molding tray filled with embedding solution, aligned using forceps, and covered with a plastic stub block holder (Indigo Scientific). Following the setting of the JB‐4, the samples were removed from the embedding mold and baked for 48 hr at 70°C to harden the blocks prior to sectioning. Blocks were stored in tightly closed and dry containers in a dry and cool place, to avoid humidity and warm temperatures which soften the blocks and prolonged exposure to ambient light which causes block surface bleaching.

### 2.5 HREM sectioning and data generation

Data generation was performed as described by Geyer et al. (2017). Briefly, blocks were mounted on the robotic microtome HREM unit (Indigo Scientific) equipped with MVX10 Olympus Microscope (Olympus) and ProgRes MFscan digital camera (Jenoptic). 2.5‐µm‐thick sections were cut, and images of the block cut surface were captured after each section with a GFP fluorescent filter (excitation filter 470/40; emission filter 525/50) at a resolution of 2720 × 2048 pixels at 16 bit. For accurate magnification calculations, an image of 1000μm graticule bar (EMS) was taken following each sample.

### 2.6 Data processing and 3D modeling

Data processing and 3D modeling were performed as described by Weninger et al., 2018. Briefly, individual images were automatically processed for leveling the pixels histogram, black and white inverted and converted to 8‐bit grayscale mode using ImageJ (Rueden et al., 2017) or Fiji (Schindelin et al., 2012) software. If section thickness did not correspond with the pixel size to form a cubic voxel, scaling of the single images to match the section thickness was done using Photoshop (Adobe Systems). Alternatively, the parameters for cubic voxel size were directly loaded to the 3D visualization software. Image stacks were loaded for 3D visualization and segmentation analysis using ImageJ Volume viewer or Amira (ThermoFisher Scientific).

## 3 RESULTS AND DISCUSSION

### 3.1 HREM 3D analysis of citrus secretory cavity development

**Figure 1:**
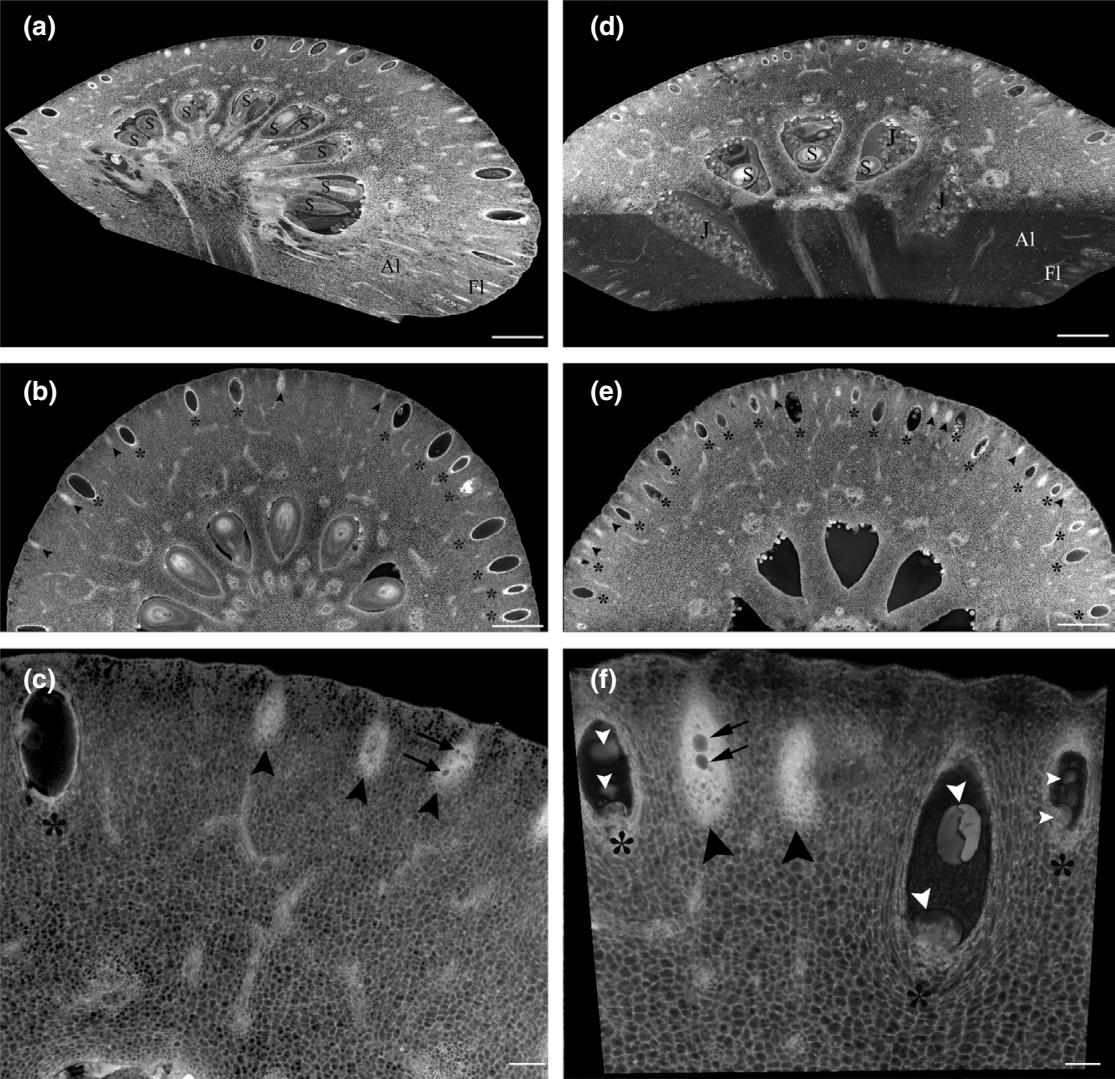
Secretory cavities in young *Citrus limon* fruitlets. 3D histology model of a 5‐mm *Citrus limon* fruitlet (a). 5‐mm fruitlet showing secretory cavities at the cell cluster stage and cavities with lumens (b). Early stages of secretory cavity initiation in 5‐mm fruitlet peel: cell clusters, cell clusters with lumen initiation, and secretory cavities with lumens (c). 3D histology model of an 8‐mm fruitlet (d). 8‐mm fruitlet showing secretory cavities at different developmental stages: cell clusters, and cavities with lumens (e). Different stages of secretory cavity initiation in 8‐mm fruitlet peel: cell clusters, cell clusters with lumen initiation, and secretory cavities with lumens containing oil droplets (f). Black arrowhead—cell cluster; black asterisk—cavity with lumen; black arrow—lumen initiation; white arrowhead—oil droplet. Bars (a‐b, d‐e) = 500 µm, (c, f) = 100 µm. For 3D models, see Movies S1–S3

**Figure 2:**
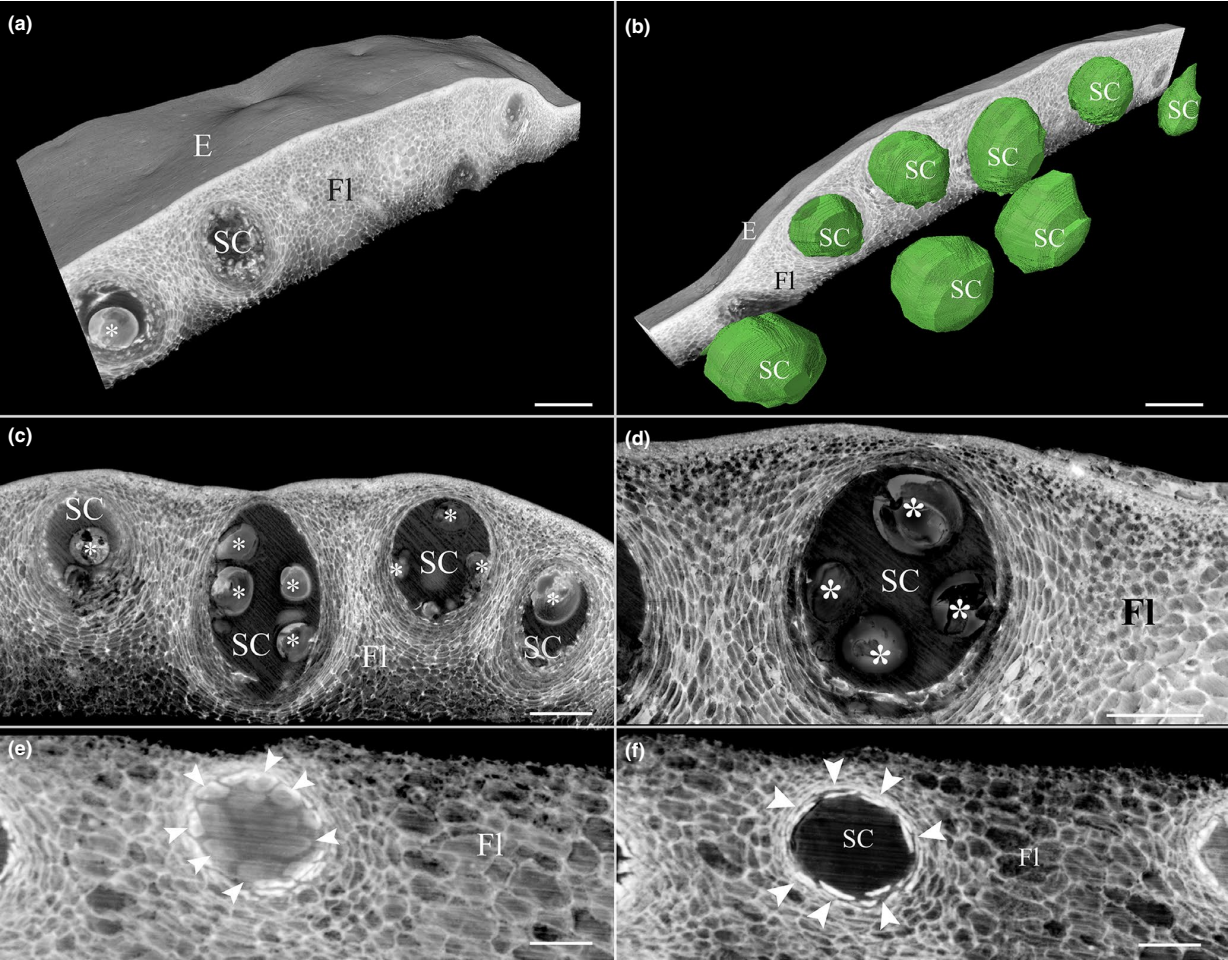
Secretory cavities in the peel of a mature *Citrus limon* fruit. 3D histology model of a mature *Citrus limon* fruit peel (a). 3D visualization of secretory cavities' shape, volume, and distribution at the mature stage (b). Mature fruit peel showing mature secretory cavities with oil droplets (c). Close up of a mature secretory cavity containing oil droplets (d). Longitudinal (e) and transverse (f) sections of the epithelial cells lining the cavity lumen. E, epidermis; Fl, flavedo; SC, secretory cavity; asterisk—oil droplet; arrowhead—epithelial cell. Bars (a–f) = 200 µm. For 3D model see Movie S5

**Figure 3:**
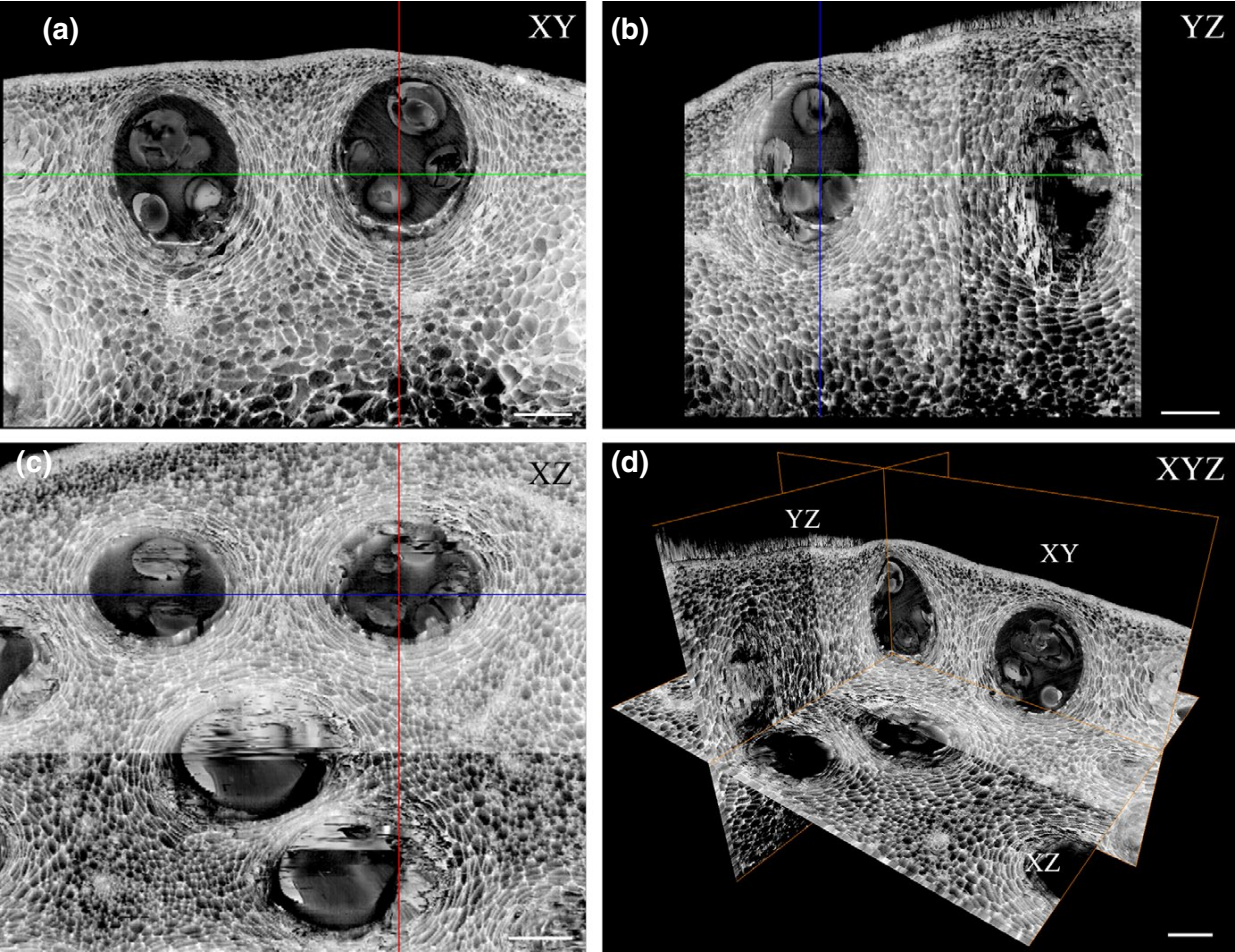
Virtual sections of secretory cavities in the peel of mature *Citrus limon* fruit. Visualization of the secretory cavities in XY (a), YZ (b), XZ (c), and XYZ (d) plains. Red line—x‐axis plane, green line—y‐axis plane, blue line—z‐axis plane. Bars (a–d) = 200 µm. For 3D model, see Movie S6

To characterize *Citrus limon* secretory cavity initiation and development, we investigated cavity anatomy during different stages of fruit development using HREM 3D imaging. Peels from 5‐, 8‐, and 15‐mm‐diameter fruitlets as well as peels from mature fruits were fixed and sectioned, and 3D histology models were generated. In 5‐ and 8‐mm fruitlets, initiation of secretory cavities was observed, reflected by sub‐epidermal cell divisions, which gave rise to elliptical cell clusters (Figure 1b,c,e,f, Movies S1–S3). At the next stage of cavity development, a lumen began to form schizogenously at the center of the cell cluster. Parallel initiation of lumens was observed in each cell cluster, which joined to generate a single lumen (Figure 1c,f, Movies S1–S3). The lumen continued to expand as the cavity developed (Figure 1c,f, Movie S3). No evidence of lysigenous events during lumen formation was observed (Figure 1c,f, Movie S3). Similarly, previous studies in citrus showed that secretory cavity initiation is restricted to the early stages of fruitlet development and is formed schizogenously (Knight et al., 2001; Liang et al., 2006). However, parallel events of cell separation leading to lumen initiation have not been described before, demonstrating the unique ability of tissue 3D visualization to capture subtle events occurring repeatedly during development. Secretory cavities at three different developmental stages were observed in 5‐ and 8‐mm fruitlets: cavities at the cell cluster stage, cell clusters with initial cell separation, and cavities with defined lumen (Figure 1b,c,e,f, Movies S1–S3). In 15‐mm fruitlets, all cavities contained a defined lumen and no cavity initiation was observed (Movie S4). Using the HREM models, essential oil droplets could be observed in the cavity lumens. Oil droplets were already detected in immature cavities in 5‐mm fruitlets and continued to accumulate as the lumen expanded (Figure 1b,c,e,f, Movies S1–S3). The different 3D HREM models demonstrate the spatial distribution of the secretory cavities in the fruit peel, featuring a single sub‐epidermal layer of secretory cavities which encompass the fruitlet circumferences (Figure 1, Movie S1, S2, S4). The 3D models generated from the different stages of fruit development enabled accurate calculation of spatial oil cavity density. Fruit development was characterized by a decrease in cavity density with 70, 57 and 24 cavities/mm^2^ in 5‐, 8‐ and 15‐mm fruitlets, respectfully, supporting the observation that secretory cavity initiation is restricted to the early stages of fruitlet development (Movie S1, S2, S4). 3D visualization of the secretory cavities at the mature stage demonstrated the cavities' elliptical egg‐like shape and revealed the cavity distribution. 3D segmentation enabled exact measurement of the cavity volumes (0.122 ± 0.021 mm^3^) (Figure 2b, Movie S5). The epithelial cells lining the cavity lumen were clearly observed (Figure 2e–f). Virtual sections of the mature stage 3D model (Movie S5) enabled visualization of the secretory cavities in any plane, which allows elaborate and accurate measurements of the secretory cavities' structure and volume (Figure 3, Movie S6).

The data presented show that the 3D models generated by the HREM technique enabled a unique spatial analysis of secretory cavity development at the cellular level, as well as accurate evaluation of oil cavity distribution, density, shape, and metric analyses.

### 3.2 Histological characterization of rooting and non‐rooting persimmon rootstock cuttings

**Figure 4:**
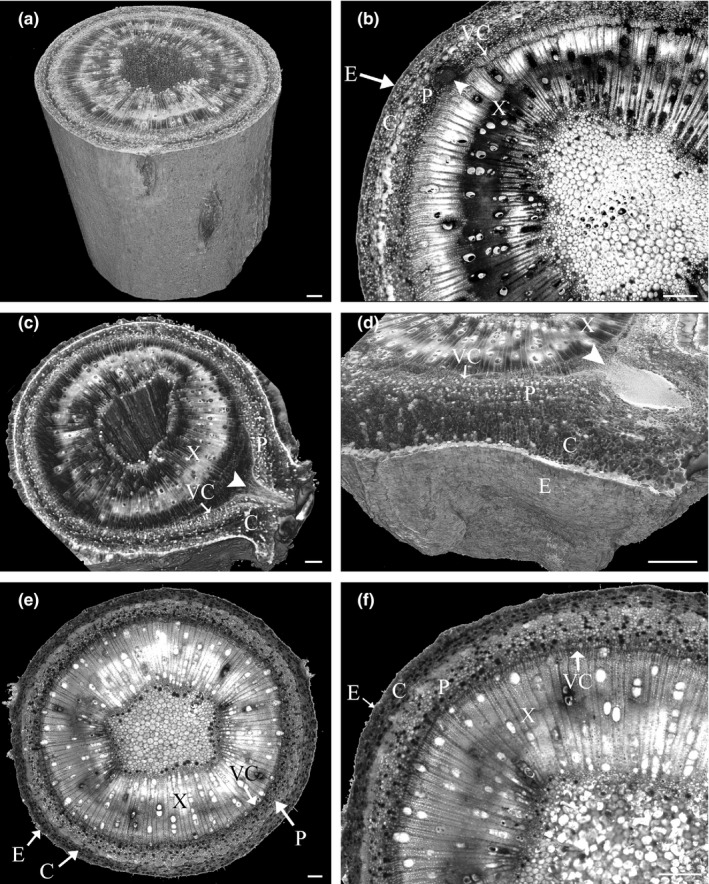
Adventitious root development in easy‐ and difficult‐to‐root persimmon (*Dyospyros virginiana*) cuttings. 3D histology model of a 10‐day‐old easy‐to‐root persimmon cutting (a). Young root primordium in a 10‐day‐old easy‐to‐root cutting (b). 3D histology model of a 20‐day‐old easy‐to‐root cutting with an adventitious root emerging from the stem cortex (c). 3D histology model close up of a root primordium emerging from the stem cortex in a 20‐day‐old easy‐to‐root cutting (d). Section through a 20‐day‐old difficult‐to‐root stem cutting (e). Close up of a 20‐day‐old difficult‐to‐root stem cutting (f). E, epidermis; C, cortex, P, phloem; VC, vascular cambium; X, xylem, arrowhead—root primordium. Bars (a–f) = 500 µm. For 3D models, see Movies S7–S10

To analyze the developmental stage at which adventitious root formation is inhibited in non‐rooting persimmon rootstocks and to explore the relationship between stem anatomy and root development, 3D histological analysis by HREM was utilized to obtain a detailed spatial visualization of the cutting bases. Easy‐ and difficult‐to‐root *D. virginiana* clones were treated with IBA and grown under controlled environment for adventitious root development. Ten and twenty days after rooting initiation, 10‐ to 15‐mm stem fragments were taken from the cutting base and sectioned by HREM. Root primordia were observed in ten‐day‐old easy‐to‐root cuttings from the phloem side of the fascicular cambium (Figure 4b, Movie S7). Twenty‐day‐old easy‐to‐root cuttings exhibited 8–10 adventitious root primordia at different stages, from young primordia to elongated roots that already emerged from the stem cortex (Figure 4c,d, Movies S8, S9) and often aligned longitudinally along the apical–basal axis of the cutting (Movie S8). The easy‐ and the difficult‐to‐root stem cuttings exhibited a similar anatomical structure (Figure 4b,f Movie S7, S10). However, while adventitious root primordia were clearly visible in the easy‐to‐root clone cuttings (Figure 4b, Movie S7), no visual signs of adventitious root primordia were observed throughout the twenty‐day‐old difficult‐to‐root stem bases (Figure 4e,f, Movie S10), suggesting that difficult‐to‐root clones fail to root due to the inhibition of root primordium initiation.

In this study, the HREM method contributed an innovative spatial characterization of the entire cutting base. The 3D models generated enabled acquisition of homologous microscopic sections of rooting and non‐rooting cuttings to allow accurate comparative anatomical analyses.

### 3.3 Prospective HREM uses

To our knowledge, the application of the HREM technique in plants is novel, providing precise spatial visualization of plant tissue anatomy and architecture. HREM systems can also be equipped with automatically revolving fluorescent filters, thus allowing for multichannel imaging. While excitation at the green channel is normally used for gross morphology, the red channel can be utilized for gene expression pattern analysis using opaque dyes such as NBT/BCIP which is commonly used for RNA in situ hybridization or X‐gal staining to detect β‐galactosidase activity. The UV channel can be applied for nuclear staining using DAPI and the far‐red wavelength for whole‐mount antibody staining. Thus, the application of HREM demonstrated here for plant research has good potential to be further developed and to provide a strong set of tools to support studies in plant development.

## CONFLICT OF INTEREST

The authors declare no conflict of interest associated with the work described in this manuscript.

## AUTHOR CONTRIBUTIONS

O.G, Y.Y., I.D., and F.S. performed experiments. A.I and J.R designed experiments. Y.C analyzed the data. A.I. drafted the manuscript with contributions from Y.C and J.R.
