# Supplementary material for: High‐resolution episcopic microscopy enables three‐dimensional visualization of plant morphology and development
Source: Plant Direct. 2019 Nov 6;3(11):e00161. doi: 10.1002/pld3.161 (PMC6834379; doi:10.1002/pld3.161)
Supplement: Supplementary file 13 [file PLD3-3-e00161-s013.docx]

A higher resolution version of this video can be obtained by contacting the Corresponding Author.
