# Supplementary material for: High‐resolution episcopic microscopy enables three‐dimensional visualization of plant morphology and development
Source: Plant Direct. 2019 Nov 6;3(11):e00161. doi: 10.1002/pld3.161 (PMC6834379; doi:10.1002/pld3.161)
Supplement: Supplementary file 11 [file PLD3-3-e00161-s011.pdf]

Plant Direct Editorial Board,

We would like to thank the reviewers for investing the time and effort to comment on the manuscript. We would appreciate the opportunity to address their comments.

Reviewer 1

Comment 1: "The authors make several claims that this is the first time that this has been shown in plants, which is not permitted under the rule of the journal. Furthermore, I am not convinced that this is the first time that this technique has been used for three dimensional imaging of plant tissues. Izhaki et al published a paper in 2018 (The Tomato BLADE ON PETIOLE and TERMINATING FLOWER Regulate Leaf Axil Patterning Along the Proximal-Distal Axes) that contained HREM images of a developing in Figure 1. Nevertheless, the technique was not the focus of that paper and especially the movies presented here show the potential of HREM to reveal 3D morphology in plants."

Response: Reviewer 1 doubted the novelty of the of the HREM technology in plants based on our recent publication (Izhaki et al., Front. Plant Sci. 9:1126. doi: 10.3389/fpls.2018.01126). However, as stated by the reviewer himself, the paper shows a single HREM image and the HREM technique is not the focus of the paper. Furthermore, the paper is lacking 3D models and does not present the potential use of HREM technique in plants. Hence, we stand behind the claim that the submitted manuscript represents a novel technology application in plants which shows the contribution and potential of the HREM system to answer scientific questions in plants. However, in light of the reviewer's comments, we toned down this statement in the revised manuscript and deleted the phrase "for the first time" and the word "novel" from the Abstract, Significance statement, Introduction and Results and Discussion sections (lines #24, 38, 85, 178, 197, 221 in the tracked changes manuscript). We added a sentence in the Results and Dissscusion stating that "To our knowledge the application of the HREM technique in plant is novel…" (line #227 in the tracked changes manuscript).

Comment 2: "The authors claim that this approach provides higher resolution than optical project tomography, but this should be quantified rather than simply asserted. I also did not understand the claimed advantage of HREM over OPT that the latter is subject to computer

generated artifacts. Can the authors provide some information about the prevalence of such artifacts in OPT?"

Our manuscript does not intend to compare the different emerging 3D modeling technologies, but rather to show the potential of HREM in plants. While we did not do any comparative work between the different 3D modeling technologies, there are significant unique technical features that make HREM an attractive approach for plant 3D imaging. A previous animal study did compare the different technologies and hence we referred to it in the Introduction section, highlighting the advantage of the HREM technology in terms of resolution compared with other technologies. In computerized tomography-based imaging systems (such as OPT and micro CT), the raw data is a set of projections which is then processed by an algorithm to create virtual sections that are later used for 3D reconstruction, leading to potential artifacts given this indirect multistep process. In contrast, the raw data in the HREM system is an actual image of thousands of perfectly aligned sections, thus obviating the need for virtualization and minimizing the potential for artifacts. We revised the paragraph in the Introduction (lines #73-81) to better explain the advantage of the HREM system over tomography-based imaging systems (such as OPT and micro CT).

Comment 3: "Finally, if this manuscript is intended to illustrate the benefit of HREM to a broader audience then that audience will, by definition, not be familiar with the technique and it would therefore be helpful to have a better comparison between the sample preparation required for HREM and the other approaches, as well as some indication of the type of equipment that would be required to set up this approach. Also, is the data processing straightforward to implement for non-users?"

Response: We elaborated on the sample preparation and equipment in the Materials and Methods section (lines #267-310 in the tracked changes manuscript).

Reviewer 2

Comment 1: "The manuscript contained no details about how the imaging platform was created, except that it was a microtome-based blockface-view design. I found suggestions in the text that the imaging platform used is a commercial product. Internet searches confirmed this. I doubted applying a commercial imaging system to plant material constituted a technical advance, but maybe if it was the first time ever, as stated, and if the application would not be

obvious to others, I might see a reason to be positive. I checked Google Scholar to see if in fact the HREM system by Indigo Scientific had been used in plants before. I immediately found this paper in Frontiers in Plant Science: https://doi.org/10.3389/fpls.2018.01126. It used the same commercial HREM imaging system."

Response: We do not see how the commercial availability of the HREM system pertains to the issue of novelty here. This is the first demonstration of the use of the HREM technique to advance scientific questions in the plant sciences. Hence the novelty presented in the manuscript is in its unique application rather than in its technical characteristics. Having the HREM system commercially available opens a vast research opportunities for many labs worldwide.

Editor:

Comment 1: "Regarding the comments to the reviewers, I agree with the notion that claiming "the first" and "novel" is not appropriate in scientific journals, especially not when highlighted extensively as it is in the current form of the manuscript. Both reviewers had issue with it (the second reviewer's concern was different but overlapping). Therefore I agree that these terms need to be toned down in the abstract, intro and results. I would suggest using the term improvement instead of novel. Or not often used in plant systems. Etc. OPT and other methods have been successfully applied to basically do the same as you show in the manuscript. I would however agree to a comment in the discussion that to your knowledge, this is method is novel in plants."

Response: We toned down this statement in the revised manuscript and erased the phrase "for the first time" and the word "novel" from the Summary, Significance statement, Introduction and Results and Discussion (lines #24, 38, 85, 178, 197, 221 in the tracked changes manuscript). We added a sentence in thr Results and Disscusion stating that "To our knowledge the application of the HREM technique in plants is novel… (line #227 in the tracked changes manuscript).

Comment 2: "Regarding the comment from the reviewer on the comparison between the different methods with HREM, I agree that is not necessary. However, to clarify, you might want to state what you wrote in your response to the reviews in the introduction. This should satisfy anyone who is not convinced HREM is better than other methods. Make sure to refer to the animal paper clearly in that section."

Response: We revised the paragraph in the Introduction (lines #73-81 in the tracked changes manuscript) to better explain the advantage of the HREM system over tomography-based imaging systems (such as OPT and micro CT). The animal papers are reffered to in the Introduction (lines #73, 83 in the tracked changes manuscript).

Comment 3: Include more detail about sample preparation, equipment used etc. You could refer in the material and methods section along the lines of: We performed the sample preparation as described by xx. Briefly, xxxx. Readers should not have to go back to other articles to have a basic grasp of the experimental details.

Response: We elaborated on the sample preparation and equipment in the Materials and Methods section (lines #267-310 in the tracked changes manuscript).

Sincerely,
Anat Izhaki
