# Supplementary material for: High‐resolution episcopic microscopy enables three‐dimensional visualization of plant morphology and development
Source: Plant Direct. 2019 Nov 6;3(11):e00161. doi: 10.1002/pld3.161 (PMC6834379; doi:10.1002/pld3.161)
Supplement: Supplementary file 12 [file PLD3-3-e00161-s012.pdf]

16-Jul-2019

Dr Anat Izhaki
Volcani Center, Agricultural Research Organization, Institute of Plant Sciences
68 HaMaccabim Road
Rishon LeZion
Israel
7505101

Manuscript No. TPJ-00476-2019
Title: High-Resolution Episcopic Microscopy (HREM) Enables Three Dimensional Visualization of Plant Morphology and Development
Authors: Cinnamon, Yuval; Genin, Olga; Yitzhak, Yiftah ; David, Israel; Shaya, Felix; Riov, Joseph ; Izhaki, Anat Corresponding Author: Anat Izhaki Handling Editor: Lee Sweetlove

Dear Dr Izhaki,

Thank you for submitting your manuscript to Plant Journal. It has been reviewed by two experts in the field. They can also be viewed, along with the editorial correspondence, in your Author Centre on our online site http://mc.manuscriptcentral.com/tpj .

Based on these reviews and my own reading of the manuscript I have come to the conclusion that your manuscript is not suitable for publication in The Plant Journal.

The central issue is that the work does not, in the opinion of the reviewers, represent a technical advance and is not novel: HREM has been used previously to image plant tissues in prior publications.

Given that the reviewers were of the opinion that the underlying data is sound, and the urgency of your need to publish this work, I would strongly recommend that you consider directly transferring your paper to our sister- sound-science-journal, Plant Direct. The reviews will also be transferred and, after editorial assessment by Plant Direct, a decision will typically be reached in 5 days or less. Full instructions are given below.

Plant Direct is an open access, sound science journal launched by the American Society of Plant Biologists and the Society for Experimental Biology and provides authors with a speedy and efficient publication process. Your paper can be directly transferred to Plant Direct and the advantage of this is that the reviews will be transferred as well resulting in a very fast process at Plant Direct, currently 7 days on average to a decision. Therefore, I would like to offer you the opportunity to transfer your paper automatically to Plant Direct's submission site. As you are transferring your paper from The Plant Journal, you will also receive a discount on the publication fees.

The transfer of your manuscript and associated submission data to Plant Direct is easy and efficient. To transfer your manuscript, please forward this decision letter to plantdirect@wiley.com

Please note that transferring does not guarantee acceptance of your manuscript, as the Editors of Plant Direct will still need to undertake due evaluation of the manuscript, however most papers transferred with reviews are accepted for publication. Once the transfer of the files and data is complete, you will receive an email request from Plant Direct asking you to log into the submission site and complete the submission.

I would like to apologise once again for the length of time that it has taken us to reach this decision. I would like to emphasise that this was not in any way due to inefficiencies in our processing of your manuscript, but rather because we had to approach very many people before we were able to find someone able to review the manuscript. While this is a common problem at this time of the year when many people are travelling, it remains the case that it has taken an unacceptably long time. I hope that this experience does not put you off considering publishing your research in TPJ in future.

Best regards,

Lee Sweetlove
Editor-in-Chief
The Plant Journal
lee.sweetlove@plants.ox.ac.uk

REVIEWER COMMENTS:

Reviewer: 1

This technical advance describes an approach to observe morphology in 3D using high-resolution episcopic microscopy and shows this method in use by studying two unrelated phenomena; the development of the oil secretory cavity in citrus and adventitious roots in persimmon. The authors make several claims that this is the first time that this has been shown in plants, which is not permitted under the rule of the journal. Furthermore, I am not convinced that this is the first time that this technique has been used for three dimensional imaging of plant tissues. Izhaki et al published a paper in 2018 (The Tomato BLADE ON PETIOLE and TERMINATING FLOWER Regulate Leaf Axil Patterning Along the Proximal-Distal Axes) that contained HREM images of a developing in Figure 1. Nevertheless, the technique was not the focus of that paper and especially the movies presented here show the potential of HREM to reveal 3D morphology in plants. The authors claim that this approach provides higher resolution than optical project tomography, but this should be quantified rather than simply asserted. I also did not understand the claimed advantage of HREM over OPT that the latter is subject to computer generated artifacts. Can the authors provide some information about the prevalence of such artifacts in OPT? Finally, if this manuscript is intended to illustrate the benefit of HREM to a broader audience then that audience will, by definition, not be familiar with the technique and it would therefore be helpful to have a better comparison between the sample preparation required for HREM and the other approaches, as well as some indication of the type of equipment that would be required to set up this approach. Also, is the data processing straightforward to implement for non-users?

The movies illustrate the potential of this approach very nicely, but are they all necessary?

Reviewer: 2

The manuscript is presented as a technological advance in 3D imaging demonstrated on two different anatomical subjects. I was expecting to learn about a novel assembly of

components to form a new methodology that would be useful for plant science research. I read about the application and the structures being studied, was impressed by the images, found the movies to give wonderful views of the anatomy, could be persuaded that the findings had intrinsic merit, but I could find no information about the technical advance. The manuscript contained no details about how the imaging platform was created, except that it was a microtome-based blockface-view design. I found suggestions in the text that the imaging platform used is a commercial product. Internet searches confirmed this. I doubted applying a commercial imaging system to plant material constituted a technical advance, but maybe if it was the first time ever, as stated, and if the application would not be obvious to others, I might see a reason to be positive. I checked Google Scholar to see if in fact the HREM system by Indigo Scientific had been used in plants before. I immediately found this paper in Frontiers in Plant Science: https://doi.org/10.3389/fpls.2018.01126. It used the same commercial HREM imaging system.

In summary -

The technical aspect of the paper is a commercial product.

It works well on plant material.

It has been used to study plant anatomy in at least one paper last year.

I do not believe that the authors can claim to have created a technical advance.

To help us improve the service that we provide to authors The Plant Journal Editorial Office would be very grateful if you could complete this short questionnaire. Your responses remain anonymous unless you choose to share your email address.

The Plant Journal is a leading journal in its field. ISI Journal Citation Reports © Ranking: 2018: 11/228 (Plant Sciences); Impact Factor: 5.726; time to first decision 29 days.

Email secured by Check Point - Threat Emulation policy
